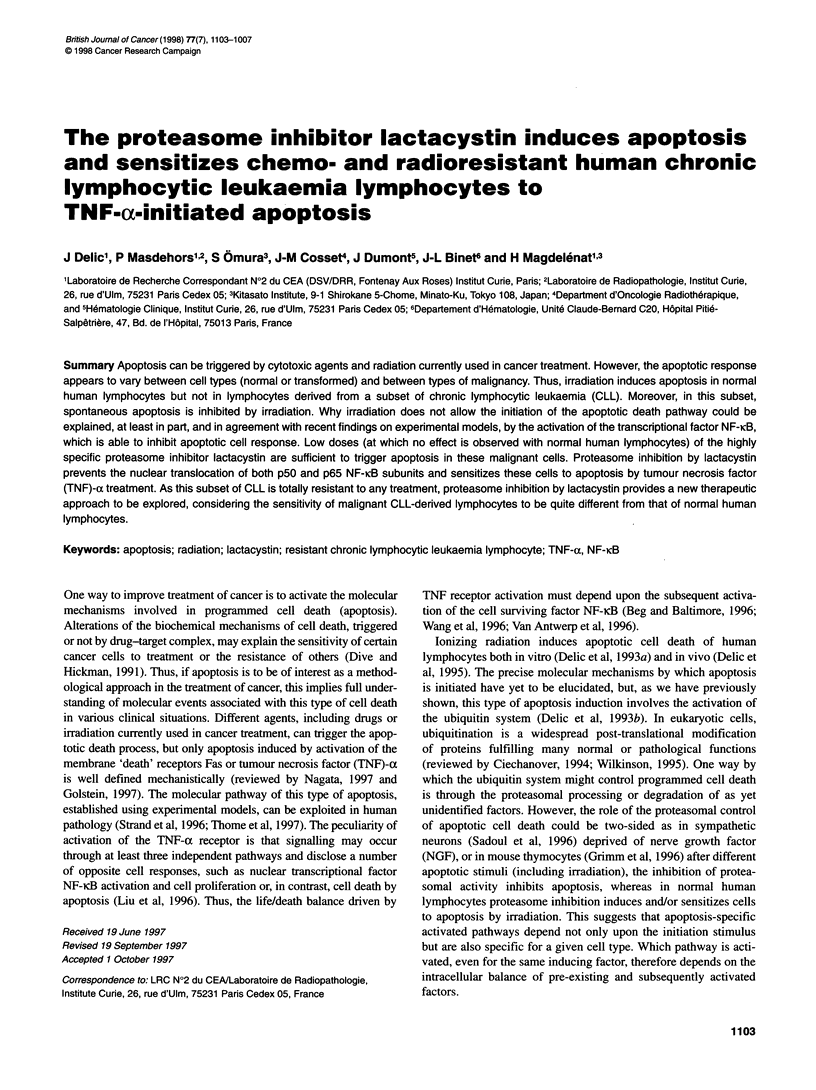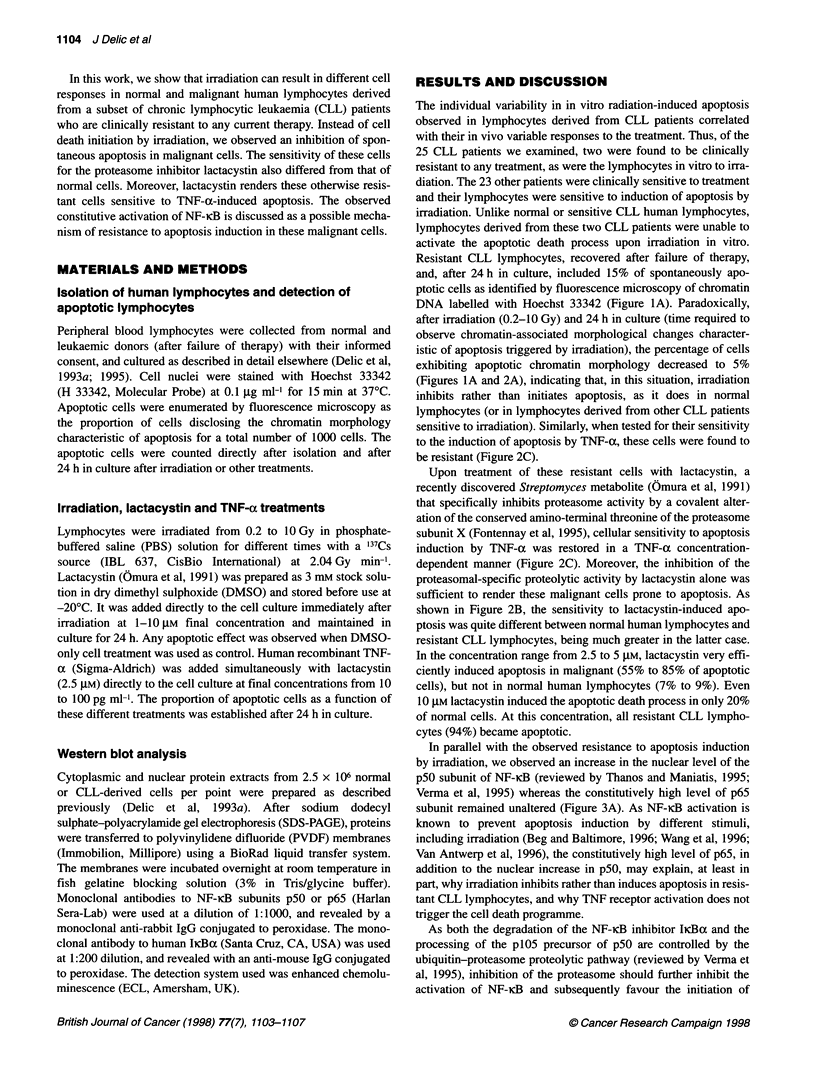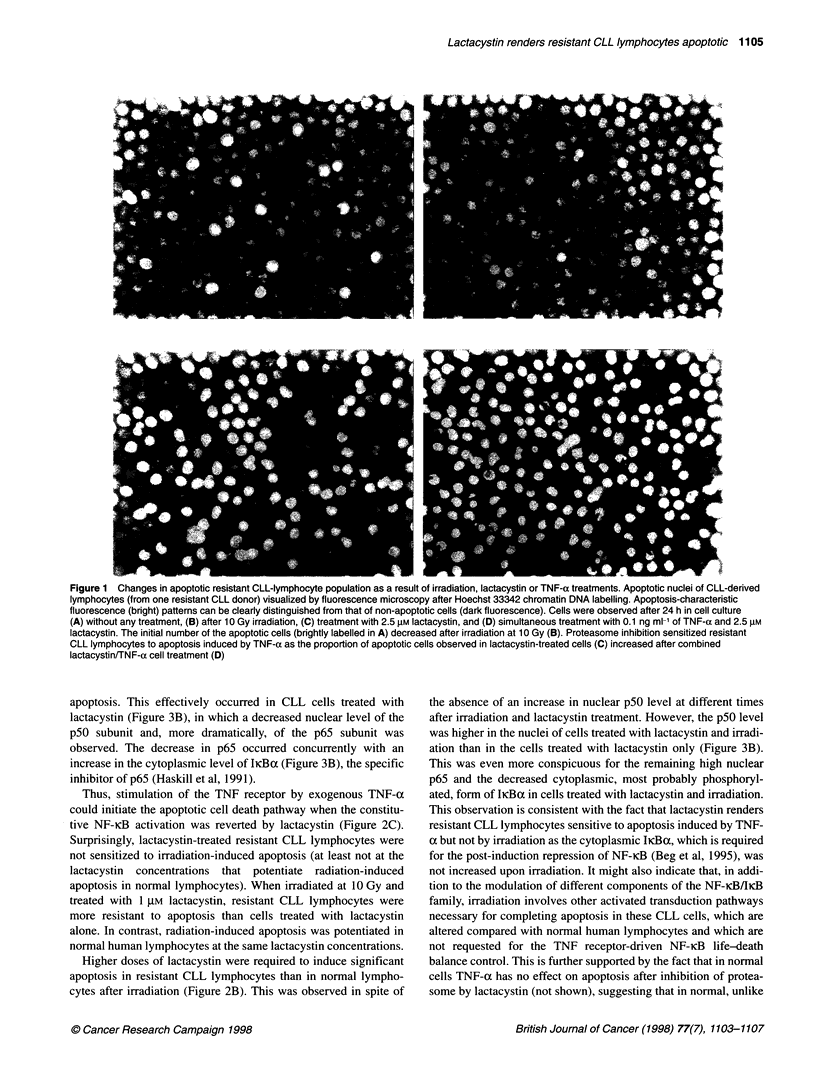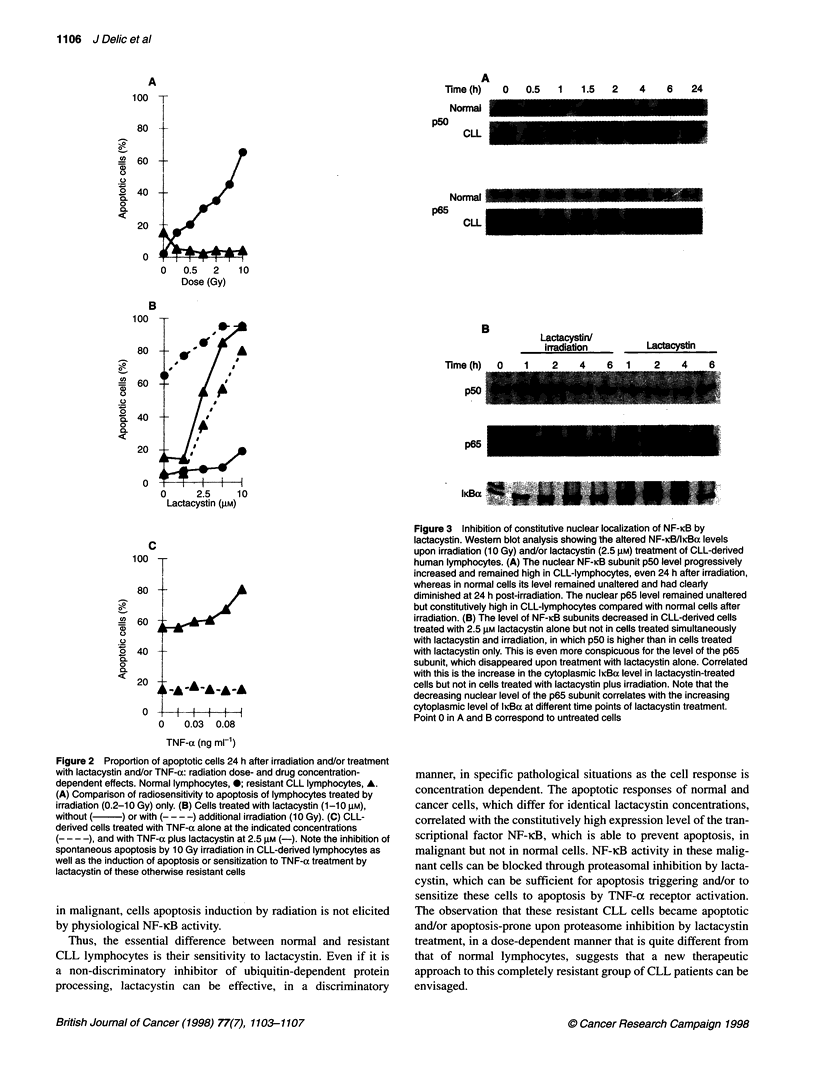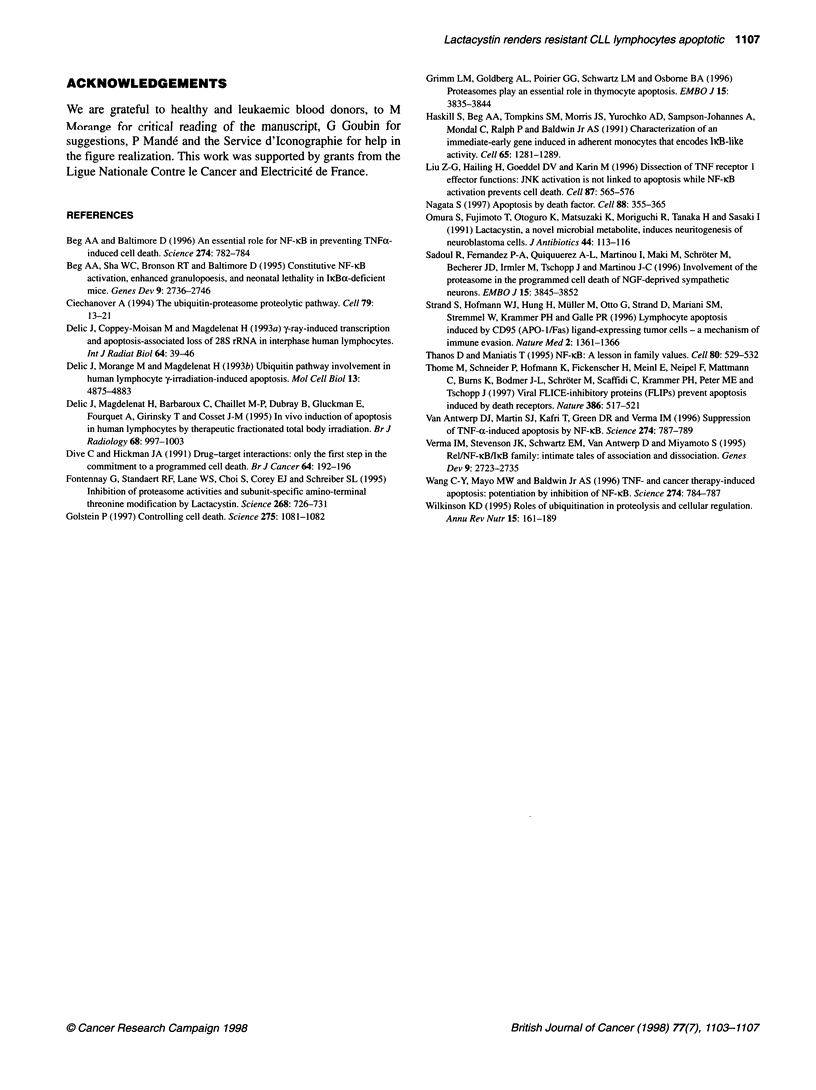# The proteasome inhibitor lactacystin induces apoptosis and sensitizes chemo- and radioresistant human chronic lymphocytic leukaemia lymphocytes to TNF-alpha-initiated apoptosis.

**DOI:** 10.1038/bjc.1998.183

**Published:** 1998-04

**Authors:** J. Delic, P. Masdehors, S. Omura, J. M. Cosset, J. Dumont, J. L. Binet, H. MagdelÃ©nat

**Affiliations:** Laboratoire de Recherche Correspondant No2 du CEA (DSV/DRR, Fontenay Aux Roses) Institut Curie, Paris, France.

## Abstract

**Images:**


British Journal of Cancer (1998) 77(7), 1103–1007
© 1998 Cancer Research Campaign

### The proteasome inhibitor lactacystin induces apoptosis and sensitizes chemo- and radioresistant human chronic lymphocytic leukaemia lymphocytes to TNF-α-initiated apoptosis

**J Delic[1], P Masdehors[1,2], S Ōmura[3], J-M Cosset[4], J Dumont[5], J-L Binet[6] and H Magdelénat[1,3]**

[1]Laboratoire de Recherche Correspondant N°2 du CEA (DSV/DRR, Fontenay Aux Roses) Institut Curie, Paris; [2]Laboratoire de Radiopathologie, Institut Curie, 26, rue d'Ulm, 75231 Paris Cedex 05; [3]Kitasato Institute, 9-1 Shirokane 5-Chome, Minato-Ku, Tokyo 108, Japan; [4]Department d'Oncologie Radiothérapique, and [5]Hématologie Clinique, Institut Curie, 26, rue d'Ulm, 75231 Paris Cedex 05; [6]Departement d'Hématologie, Unité Claude-Bernard C20, Hôpital Pitié-Salpêtrière, 47, Bd. de l'Hôpital, 75013 Paris, France

**Summary** Apoptosis can be triggered by cytotoxic agents and radiation currently used in cancer treatment. However, the apoptotic response appears to vary between cell types (normal or transformed) and between types of malignancy. Thus, irradiation induces apoptosis in normal human lymphocytes but not in lymphocytes derived from a subset of chronic lymphocytic leukaemia (CLL). Moreover, in this subset, spontaneous apoptosis is inhibited by irradiation. Why irradiation does not allow the initiation of the apoptotic death pathway could be explained, at least in part, and in agreement with recent findings on experimental models, by the activation of the transcriptional factor NF-κB, which is able to inhibit apoptotic cell response. Low doses (at which no effect is observed with normal human lymphocytes) of the highly specific proteasome inhibitor lactacystin are sufficient to trigger apoptosis in these malignant cells. Proteasome inhibition by lactacystin prevents the nuclear translocation of both p50 and p65 NF-κB subunits and sensitizes these cells to apoptosis by tumour necrosis factor (TNF)-α treatment. As this subset of CLL is totally resistant to any treatment, proteasome inhibition by lactacystin provides a new therapeutic approach to be explored, considering the sensitivity of malignant CLL-derived lymphocytes to be quite different from that of normal human lymphocytes.

**Keywords:** apoptosis; radiation; lactacystin; resistant chronic lymphocytic leukaemia lymphocyte; TNF-α, NF-κB

One way to improve treatment of cancer is to activate the molecular mechanisms involved in programmed cell death (apoptosis). Alterations of the biochemical mechanisms of cell death, triggered or not by drug–target complex, may explain the sensitivity of certain cancer cells to treatment or the resistance of others (Dive and Hickman, 1991). Thus, if apoptosis is to be of interest as a methodological approach in the treatment of cancer, this implies full understanding of molecular events associated with this type of cell death in various clinical situations. Different agents, including drugs or irradiation currently used in cancer treatment, can trigger the apoptotic death process, but only apoptosis induced by activation of the membrane 'death' receptors Fas or tumour necrosis factor (TNF)-α is well defined mechanistically (reviewed by Nagata, 1997 and Golstein, 1997). The molecular pathway of this type of apoptosis, established using experimental models, can be exploited in human pathology (Strand et al, 1996; Thome et al, 1997). The peculiarity of activation of the TNF-α receptor is that signalling may occur through at least three independent pathways and disclose a number of opposite cell responses, such as nuclear transcriptional factor NF-κB activation and cell proliferation or, in contrast, cell death by apoptosis (Liu et al, 1996). Thus, the life/death balance driven by TNF receptor activation must depend upon the subsequent activation of the cell surviving factor NF-κB (Beg and Baltimore, 1996; Wang et al, 1996; Van Antwerp et al, 1996).

*Received 19 June 1997*
*Revised 19 September 1997*
*Accepted 1 October 1997*

*Correspondence to:* LRC N°2 du CEA/Laboratoire de Radiopathologie, Institute Curie, 26, rue d'Ulm, 75231 Paris Cedex 05, France

Ionizing radiation induces apoptotic cell death of human lymphocytes both in vitro (Delic et al, 1993*a*) and in vivo (Delic et al, 1995). The precise molecular mechanisms by which apoptosis is initiated have yet to be elucidated, but, as we have previously shown, this type of apoptosis induction involves the activation of the ubiquitin system (Delic et al, 1993*b*). In eukaryotic cells, ubiquitination is a widespread post-translational modification of proteins fulfilling many normal or pathological functions (reviewed by Ciechanover, 1994; Wilkinson, 1995). One way by which the ubiquitin system might control programmed cell death is through the proteasomal processing or degradation of as yet unidentified factors. However, the role of the proteasomal control of apoptotic cell death could be two-sided as in sympathetic neurons (Sadoul et al, 1996) deprived of nerve growth factor (NGF), or in mouse thymocytes (Grimm et al, 1996) after different apoptotic stimuli (including irradiation), the inhibition of proteasomal activity inhibits apoptosis, whereas in normal human lymphocytes proteasome inhibition induces and/or sensitizes cells to apoptosis by irradiation. This suggests that apoptosis-specific activated pathways depend not only upon the initiation stimulus but are also specific for a given cell type. Which pathway is activated, even for the same inducing factor, therefore depends on the intracellular balance of pre-existing and subsequently activated factors.

In this work, we show that irradiation can result in different cell responses in normal and malignant human lymphocytes derived from a subset of chronic lymphocytic leukaemia (CLL) patients who are clinically resistant to any current therapy. Instead of cell death initiation by irradiation, we observed an inhibition of spontaneous apoptosis in malignant cells. The sensitivity of these cells for the proteasome inhibitor lactacystin also differed from that of normal cells. Moreover, lactacystin renders these otherwise resistant cells sensitive to TNF-α-induced apoptosis. The observed constitutive activation of NF-κB is discussed as a possible mechanism of resistance to apoptosis induction in these malignant cells.

### MATERIALS AND METHODS

### Isolation of human lymphocytes and detection of apoptotic lymphocytes

Peripheral blood lymphocytes were collected from normal and leukaemic donors (after failure of therapy) with their informed consent, and cultured as described in detail elsewhere (Delic et al, 1993*a*; 1995). Cell nuclei were stained with Hoechst 33342 (H 33342, Molecular Probe) at 0.1 μg $ml^{-1}$ for 15 min at 37°C. Apoptotic cells were enumerated by fluorescence microscopy as the proportion of cells disclosing the chromatin morphology characteristic of apoptosis for a total number of 1000 cells. The apoptotic cells were counted directly after isolation and after 24 h in culture after irradiation or other treatments.

### Irradiation, lactacystin and TNF-α treatments

Lymphocytes were irradiated from 0.2 to 10 Gy in phosphate-buffered saline (PBS) solution for different times with a $^{137}Cs$ source (IBL 637, CisBio International) at 2.04 Gy $min^{-1}$. Lactacystin (Ömura et al, 1991) was prepared as 3 mM stock solution in dry dimethyl sulphoxide (DMSO) and stored before use at –20°C. It was added directly to the cell culture immediately after irradiation at 1–10 μM final concentration and maintained in culture for 24 h. Any apoptotic effect was observed when DMSO-only cell treatment was used as control. Human recombinant TNF-α (Sigma-Aldrich) was added simultaneously with lactacystin (2.5 μM) directly to the cell culture at final concentrations from 10 to 100 pg $ml^{-1}$. The proportion of apoptotic cells as a function of these different treatments was established after 24 h in culture.

### Western blot analysis

Cytoplasmic and nuclear protein extracts from $2.5 \times 10^6$ normal or CLL-derived cells per point were prepared as described previously (Delic et al, 1993*a*). After sodium dodecyl sulphate–polyacrylamide gel electrophoresis (SDS-PAGE), proteins were transferred to polyvinylidene difluoride (PVDF) membranes (Immobilion, Millipore) using a BioRad liquid transfer system. The membranes were incubated overnight at room temperature in fish gelatine blocking solution (3% in Tris/glycine buffer). Monoclonal antibodies to NF-κB subunits p50 or p65 (Harlan Sera-Lab) were used at a dilution of 1:1000, and revealed by a monoclonal anti-rabbit IgG conjugated to peroxidase. The monoclonal antibody to human IκBα (Santa Cruz, CA, USA) was used at 1:200 dilution, and revealed with an anti-mouse IgG conjugated to peroxidase. The detection system used was enhanced chemoluminescence (ECL, Amersham, UK).

### RESULTS AND DISCUSSION

The individual variability in in vitro radiation-induced apoptosis observed in lymphocytes derived from CLL patients correlated with their in vivo variable responses to the treatment. Thus, of the 25 CLL patients we examined, two were found to be clinically resistant to any treatment, as were the lymphocytes in vitro to irradiation. The 23 other patients were clinically sensitive to treatment and their lymphocytes were sensitive to induction of apoptosis by irradiation. Unlike normal or sensitive CLL human lymphocytes, lymphocytes derived from these two CLL patients were unable to activate the apoptotic death process upon irradiation in vitro. Resistant CLL lymphocytes, recovered after failure of therapy, and, after 24 h in culture, included 15% of spontaneously apoptotic cells as identified by fluorescence microscopy of chromatin DNA labelled with Hoechst 33342 (Figure 1A). Paradoxically, after irradiation (0.2–10 Gy) and 24 h in culture (time required to observe chromatin-associated morphological changes characteristic of apoptosis triggered by irradiation), the percentage of cells exhibiting apoptotic chromatin morphology decreased to 5% (Figures 1A and 2A), indicating that, in this situation, irradiation inhibits rather than initiates apoptosis, as it does in normal lymphocytes (or in lymphocytes derived from other CLL patients sensitive to irradiation). Similarly, when tested for their sensitivity to the induction of apoptosis by TNF-α, these cells were found to be resistant (Figure 2C).

Upon treatment of these resistant cells with lactacystin, a recently discovered *Streptomyces* metabolite (Ömura et al, 1991) that specifically inhibits proteasome activity by a covalent alteration of the conserved amino-terminal threonine of the proteasome subunit X (Fontennay et al, 1995), cellular sensitivity to apoptosis induction by TNF-α was restored in a TNF-α concentration-dependent manner (Figure 2C). Moreover, the inhibition of the proteasomal-specific proteolytic activity by lactacystin alone was sufficient to render these malignant cells prone to apoptosis. As shown in Figure 2B, the sensitivity to lactacystin-induced apoptosis was quite different between normal human lymphocytes and resistant CLL lymphocytes, being much greater in the latter case. In the concentration range from 2.5 to 5 μM, lactacystin very efficiently induced apoptosis in malignant (55% to 85% of apoptotic cells), but not in normal human lymphocytes (7% to 9%). Even 10 μM lactacystin induced the apoptotic death process in only 20% of normal cells. At this concentration, all resistant CLL lymphocytes (94%) became apoptotic.

In parallel with the observed resistance to apoptosis induction by irradiation, we observed an increase in the nuclear level of the p50 subunit of NF-κB (reviewed by Thanos and Maniatis, 1995; Verma et al, 1995) whereas the constitutively high level of p65 subunit remained unaltered (Figure 3A). As NF-κB activation is known to prevent apoptosis induction by different stimuli, including irradiation (Beg and Baltimore, 1996; Wang et al, 1996; Van Antwerp et al, 1996), the constitutively high level of p65, in addition to the nuclear increase in p50, may explain, at least in part, why irradiation inhibits rather than induces apoptosis in resistant CLL lymphocytes, and why TNF receptor activation does not trigger the cell death programme.

As both the degradation of the NF-κB inhibitor IκBα and the processing of the p105 precursor of p50 are controlled by the ubiquitin–proteasome proteolytic pathway (reviewed by Verma et al, 1995), inhibition of the proteasome should further inhibit the activation of NF-κB and subsequently favour the initiation of

 © Cancer Research Campaign 1998

**Figure 1** Changes in apoptotic resistant CLL-lymphocyte population as a result of irradiation, lactacystin or TNF-α treatments. Apoptotic nuclei of CLL-derived lymphocytes (from one resistant CLL donor) visualized by fluorescence microscopy after Hoechst 33342 chromatin DNA labelling. Apoptosis-characteristic fluorescence (bright) patterns can be clearly distinguished from that of non-apoptotic cells (dark fluorescence). Cells were observed after 24 h in cell culture (**A**) without any treatment, (**B**) after 10 Gy irradiation, (**C**) treatment with 2.5 μM lactacystin, and (**D**) simultaneous treatment with 0.1 ng $ml^{-1}$ of TNF-α and 2.5 μM lactacystin. The initial number of the apoptotic cells (brightly labelled in **A**) decreased after irradiation at 10 Gy (**B**). Proteasome inhibition sensitized resistant CLL lymphocytes to apoptosis induced by TNF-α as the proportion of apoptotic cells observed in lactacystin-treated cells (**C**) increased after combined lactacystin/TNF-α cell treatment (**D**)

apoptosis. This effectively occurred in CLL cells treated with lactacystin (Figure 3B), in which a decreased nuclear level of the p50 subunit and, more dramatically, of the p65 subunit was observed. The decrease in p65 occurred concurrently with an increase in the cytoplasmic level of IκBα (Figure 3B), the specific inhibitor of p65 (Haskill et al, 1991).

Thus, stimulation of the TNF receptor by exogenous TNF-α could initiate the apoptotic cell death pathway when the constitutive NF-κB activation was reverted by lactacystin (Figure 2C). Surprisingly, lactacystin-treated resistant CLL lymphocytes were not sensitized to irradiation-induced apoptosis (at least not at the lactacystin concentrations that potentiate radiation-induced apoptosis in normal lymphocytes). When irradiated at 10 Gy and treated with 1 μM lactacystin, resistant CLL lymphocytes were more resistant to apoptosis than cells treated with lactacystin alone. In contrast, radiation-induced apoptosis was potentiated in normal human lymphocytes at the same lactacystin concentrations.

Higher doses of lactacystin were required to induce significant apoptosis in resistant CLL lymphocytes than in normal lymphocytes after irradiation (Figure 2B). This was observed in spite of the absence of an increase in nuclear p50 level at different times after irradiation and lactacystin treatment. However, the p50 level was higher in the nuclei of cells treated with lactacystin and irradiation than in the cells treated with lactacystin only (Figure 3B). This was even more conspicuous for the remaining high nuclear p65 and the decreased cytoplasmic, most probably phosphorylated, form of IκBα in cells treated with lactacystin and irradiation. This observation is consistent with the fact that lactacystin renders resistant CLL lymphocytes sensitive to apoptosis induced by TNF-α but not by irradiation as the cytoplasmic IκBα, which is required for the post-induction repression of NF-κB (Beg et al, 1995), was not increased upon irradiation. It might also indicate that, in addition to the modulation of different components of the NF-κB/IκB family, irradiation involves other activated transduction pathways necessary for completing apoptosis in these CLL cells, which are altered compared with normal human lymphocytes and which are not requested for the TNF receptor-driven NF-κB life–death balance control. This is further supported by the fact that in normal cells TNF-α has no effect on apoptosis after inhibition of proteasome by lactacystin (not shown), suggesting that in normal, unlike

© Cancer Research Campaign 1998

**Figure 2** Proportion of apoptotic cells 24 h after irradiation and/or treatment with lactacystin and/or TNF-α: radiation dose- and drug concentration-dependent effects. Normal lymphocytes, ●; resistant CLL lymphocytes, ▲. (**A**) Comparison of radiosensitivity to apoptosis of lymphocytes treated by irradiation (0.2–10 Gy) only. (**B**) Cells treated with lactacystin (1–10 μM), without (———) or with (- - - -) additional irradiation (10 Gy). (**C**) CLL-derived cells treated with TNF-α alone at the indicated concentrations (- - - -), and with TNF-α plus lactacystin at 2.5 μM (—). Note the inhibition of spontaneous apoptosis by 10 Gy irradiation in CLL-derived lymphocytes as well as the induction of apoptosis or sensitization to TNF-α treatment by lactacystin of these otherwise resistant cells

**Figure 3** Inhibition of constitutive nuclear localization of NF-κB by lactacystin. Western blot analysis showing the altered NF-κB/IκBα levels upon irradiation (10 Gy) and/or lactacystin (2.5 μM) treatment of CLL-derived human lymphocytes. (**A**) The nuclear NF-κB subunit p50 level progressively increased and remained high in CLL-lymphocytes, even 24 h after irradiation, whereas in normal cells its level remained unaltered and had clearly diminished at 24 h post-irradiation. The nuclear p65 level remained unaltered but constitutively high in CLL-lymphocytes compared with normal cells after irradiation. (**B**) The level of NF-κB subunits decreased in CLL-derived cells treated with 2.5 μM lactacystin alone but not in cells treated simultaneously with lactacystin and irradiation, in which p50 is higher than in cells treated with lactacystin only. This is even more conspicuous for the level of the p65 subunit, which disappeared upon treatment with lactacystin alone. Correlated with this is the increase in the cytoplasmic IκBα level in lactacystin-treated cells but not in cells treated with lactacystin plus irradiation. Note that the decreasing nuclear level of the p65 subunit correlates with the increasing cytoplasmic level of IκBα at different time points of lactacystin treatment. Point 0 in A and B correspond to untreated cells in malignant, cells apoptosis induction by radiation is not elicited by physiological NF-κB activity.

Thus, the essential difference between normal and resistant CLL lymphocytes is their sensitivity to lactacystin. Even if it is a non-discriminatory inhibitor of ubiquitin-dependent protein processing, lactacystin can be effective, in a discriminatory manner, in specific pathological situations as the cell response is concentration dependent. The apoptotic responses of normal and cancer cells, which differ for identical lactacystin concentrations, correlated with the constitutively high expression level of the transcriptional factor NF-κB, which is able to prevent apoptosis, in malignant but not in normal cells. NF-κB activity in these malignant cells can be blocked through proteasomal inhibition by lactacystin, which can be sufficient for apoptosis triggering and/or to sensitize these cells to apoptosis by TNF-α receptor activation. The observation that these resistant CLL cells became apoptotic and/or apoptosis-prone upon proteasome inhibition by lactacystin treatment, in a dose-dependent manner that is quite different from that of normal lymphocytes, suggests that a new therapeutic approach to this completely resistant group of CLL patients can be envisaged.

© Cancer Research Campaign 1998

### ACKNOWLEDGEMENTS

We are grateful to healthy and leukaemic blood donors, to M Morange for critical reading of the manuscript, G Goubin for suggestions, P Mandé and the Service d'Iconographie for help in the figure realization. This work was supported by grants from the Ligue Nationale Contre le Cancer and Electricité de France.

### REFERENCES

Beg AA and Baltimore D (1996) An essential role for NF-κB in preventing TNFα-induced cell death. *Science* **274**: 782–784

Beg AA, Sha WC, Bronson RT and Baltimore D (1995) Constitutive NF-κB activation, enhanced granulopoesis, and neonatal lethality in IκBα-deficient mice. *Genes Dev* **9**: 2736–2746

Ciechanover A (1994) The ubiquitin-proteasome proteolytic pathway. *Cell* **79**: 13–21

Delic J, Coppey-Moisan M and Magdelenat H (1993*a*) γ-ray-induced transcription and apoptosis-associated loss of 28S rRNA in interphase human lymphocytes. *Int J Radiat Biol* **64**: 39–46

Delic J, Morange M and Magdelenat H (1993*b*) Ubiquitin pathway involvement in human lymphocyte γ-irradiation-induced apoptosis. *Mol Cell Biol* **13**: 4875–4883

Delic J, Magdelenat H, Barbaroux C, Chaillet M-P, Dubray B, Gluckman E, Fourquet A, Girinsky T and Cosset J-M (1995) In vivo induction of apoptosis in human lymphocytes by therapeutic fractionated total body irradiation. *Br J Radiology* **68**: 997–1003

Dive C and Hickman JA (1991) Drug-target interactions: only the first step in the commitment to a programmed cell death. *Br J Cancer* **64**: 192–196

Fontennay G, Standaert RF, Lane WS, Choi S, Corey EJ and Schreiber SL (1995) Inhibition of proteasome activities and subunit-specific amino-terminal threonine modification by Lactacystin. *Science* **268**: 726–731

Golstein P (1997) Controlling cell death. *Science* **275**: 1081–1082

Grimm LM, Goldberg AL, Poirier GG, Schwartz LM and Osborne BA (1996) Proteasomes play an essential role in thymocyte apoptosis. *EMBO J* **15**: 3835–3844

Haskill S, Beg AA, Tompkins SM, Morris JS, Yurochko AD, Sampson-Johannes A, Mondal C, Ralph P and Baldwin Jr AS (1991) Characterization of an immediate-early gene induced in adherent monocytes that encodes IκB-like activity. *Cell* **65**: 1281–1289.

Liu Z-G, Hailing H, Goeddel DV and Karin M (1996) Dissection of TNF receptor 1 effector functions: JNK activation is not linked to apoptosis while NF-κB activation prevents cell death. *Cell* **87**: 565–576

Nagata S (1997) Apoptosis by death factor. *Cell* **88**: 355–365

Omura S, Fujimoto T, Otoguro K, Matsuzaki K, Moriguchi R, Tanaka H and Sasaki I (1991) Lactacystin, a novel microbial metabolite, induces neuritogenesis of neuroblastoma cells. *J Antibiotics* **44**: 113–116

Sadoul R, Fernandez P-A, Quiquuerez A-L, Martinou I, Maki M, Schröter M, Becherer JD, Irmler M, Tschopp J and Martinou J-C (1996) Involvement of the proteasome in the programmed cell death of NGF-deprived sympathetic neurons. *EMBO J* **15**: 3845–3852

Strand S, Hofmann WJ, Hung H, Müller M, Otto G, Strand D, Mariani SM, Stremmel W, Krammer PH and Galle PR (1996) Lymphocyte apoptosis induced by CD95 (APO-1/Fas) ligand-expressing tumor cells – a mechanism of immune evasion. *Nature Med* **2**: 1361–1366

Thanos D and Maniatis T (1995) NF-κB: A lesson in family values. *Cell* **80**: 529–532

Thome M, Schneider P, Hofmann K, Fickenscher H, Meinl E, Neipel F, Mattmann C, Burns K, Bodmer J-L, Schröter M, Scaffidi C, Krammer PH, Peter ME and Tschopp J (1997) Viral FLICE-inhibitory proteins (FLIPs) prevent apoptosis induced by death receptors. *Nature* **386**: 517–521

Van Antwerp DJ, Martin SJ, Kafri T, Green DR and Verma IM (1996) Suppression of TNF-α-induced apoptosis by NF-κB. *Science* **274**: 787–789

Verma IM, Stevenson JK, Schwartz EM, Van Antwerp D and Miyamoto S (1995) Rel/NF-κB/IκB family: intimate tales of association and dissociation. *Genes Dev* **9**: 2723–2735

Wang C-Y, Mayo MW and Baldwin Jr AS (1996) TNF- and cancer therapy-induced apoptosis: potentiation by inhibition of NF-κB. *Science* **274**: 784–787

Wilkinson KD (1995) Roles of ubiquitination in proteolysis and cellular regulation. *Annu Rev Nutr* **15**: 161–189

© Cancer Research Campaign 1998